# Preclinical Three-Dimensional Vibrational Shear Wave Elastography for Mapping of Tumour Biomechanical Properties In Vivo

**DOI:** 10.3390/cancers14194832

**Published:** 2022-10-03

**Authors:** Vaideesh Parasaram, John Civale, Jeffrey C. Bamber, Simon P. Robinson, Yann Jamin, Emma Harris

**Affiliations:** Division of Radiotherapy and Imaging, Centre for Cancer Imaging, Institute of Cancer Research, London SM2 5NG, UK

**Keywords:** biomechanical properties, elastography, tumour response, preclinical imaging

## Abstract

**Simple Summary:**

The increased tissue stiffness associated with cancer has been found to be a significant barrier to effective treatment and indicates an increased likelihood of cancer progression. Techniques to map tumour stiffness throughout the whole tumour in three dimensions will assist with preclinical research that aims to understand the relationship between the stiffness, the underlying tumour biology, and the response of tumours to therapy. We have developed an approach that measures the tumour stiffness in murine models of cancer, which are commonly used for cancer research. This technique uses a high-frequency vibrational source, ultrasound imaging and a three-dimensional analysis, which has advantages in terms of spatial resolution and rapid acquisition times. Here we present the first successful demonstration of the non-invasive three-dimensional measurement of tumour stiffness in two preclinical tumour models and the ability of the technique to detect a change in tumour stiffness in response to an anti-cancer drug.

**Abstract:**

Preclinical investigation of the biomechanical properties of tissues and their treatment-induced changes are essential to support drug-discovery, clinical translation of biomarkers of treatment response, and studies of mechanobiology. Here we describe the first use of preclinical 3D elastography to map the shear wave speed (c_s_), which is related to tissue stiffness, in vivo and demonstrate the ability of our novel 3D vibrational shear wave elastography (3D-VSWE) system to detect tumour response to a therapeutic challenge. We investigate the use of one or two vibrational sources at vibrational frequencies of 700, 1000 and 1200 Hz. The within-subject coefficients of variation of our system were found to be excellent for 700 and 1000 Hz and 5.4 and 6.2%, respectively. The relative change in c_s_ measured with our 3D-VSWE upon treatment with an anti-vascular therapy ZD6126 in two tumour xenografts reflected changes in tumour necrosis. U-87 MG drug vs vehicle: Δc_s_ = −24.7 ± 2.5 % vs 7.5 ± 7.1%, (*p* = 0.002) and MDA-MB-231 drug vs vehicle: Δc_s_ = −12.3 ± 2.7 % vs 4.5 ± 4.7%, (*p* = 0.02). Our system enables rapid (<5 min were required for a scan length of 15 mm and three vibrational frequencies) 3D mapping of quantitative tumour viscoelastic properties in vivo, allowing exploration of regional heterogeneity within tumours and speedy recovery of animals from anaesthesia so that longitudinal studies (e.g., during tumour growth or following treatment) may be conducted frequently.

## 1. Introduction

Mounting evidence supports the role of increased tissue stiffness in malignant transformation, tumour progression and metastasis [1,2,3,4]. Mechanical stress associated with rapid tissue growth, compressed vasculature and lymphatics, and extracellular matrix (ECM) structure and rigidity is the major contributor to this phenomenon. Both elevated solid stress and interstitial fluid pressure (IFP), which influence tumour viscoelastic properties, are two major obstacles to efficient drug delivery, and consequently, therapies targeting the stromal component of tumours are being investigated for therapeutic gain [5].

Innovative elastography techniques based on the use of ultrasound, magnetic resonance (MR) and optical imaging are being developed to non-invasively visualise and measure the viscoelastic properties of tissue in preclinical tumour models and cancer patients in vivo. These emerging methods can inform on the underlying tumour microstructure and treatment-induced changes to tumour integrity with direct clinical translation through the development and validation of biomarkers that will enhance decision-making in the oncology clinic, including for diagnosis, treatment planning and treatment response assessment [6,7,8,9,10,11,12,13,14,15,16]. They can also be expected to enhance our understanding of tumour mechanobiology and identify key genomic and pathophysiological drivers of tumour stiffness [17]. To support these efforts, there is a need for a dedicated small animal elastography platform that enables quantitative and reproducible mapping of the biomechanical properties (elastic and viscous moduli) to monitor their acute and long-term evolution upon disease progression or treatment. Rapid 3D mapping of these properties will allow exploration of regional heterogeneity within tumours and time-efficient animal procedures so that longitudinal studies (e.g., during tumour growth or following treatment) may be conducted with excellent temporal sampling. 

Shear wave elastography (SWE), which is becoming widely used in the clinic as a diagnostic tool, relies on the generation of shear waves and the subsequent rapid measurement of shear wave speed to enable the quantitative assessments of elastic and viscous moduli [18]. Measuring the shear wave speed at multiple shear wave frequencies allows the characterisation of tissue viscous modulus through measurement of the shear wave speed dispersion [18]. The measurement of the biomechanical properties of tissue enabled by ultrasound is an attractive imaging option for a high-throughput preclinical shear wave elastography platform. Commercial clinical scanners typically generate shear waves using an acoustic radiation force impulse push delivered inside the tissue. This uses a broadband transient shear wave pulse with a frequency spectrum that varies inside the tissue and depends on factors such as the depth of the push location, the tissue properties at the push location and the distance the shear waves have propagated. How these biases are corrected for by the scanner manufacturer results in variation in shear wave speed measurements for different depths and between ultrasound SWE systems [19,20,21,22]. To overcome these issues, an external continuous vibrational source can be used to generate narrow-band shear waves with controlled frequency and wave amplitude [23]. This is the approach adopted in the development of both clinical [24] and preclinical [25] magnetic resonance elastography and is also being explored for clinical use with clinical ultrasound systems [26]. 

Here, we describe the development of a three-dimensional preclinical high-frequency vibrational shear wave elastography (3D-VSWE) system, employing up to two external mechanical vibrational sources to generate continuous travelling shear waves in tissue instead of acoustic radiation force impulses. The attenuation of shear waves increases with frequency. However, since mice tumours are small, with propagation distances typically less than 20 mm, there is an opportunity to use external mechanical sources of high-frequency shear waves to achieve the spatial resolution required to investigate tumour heterogeneity and to avoid shear wave reflections from tissues beyond the tumour [27]. Because higher frequency shear waves are more rapidly attenuated, a delicate balance exists between using higher frequencies for high spatial resolution and maintaining the propagation of shear waves throughout the tumour. To help achieve this, we have developed and validated two real-time metrics of the shear wave field quality within a tumour to allow users to rapidly optimise the arrangement of vibrational sources and their frequencies and amplitude. We demonstrate that our 3D-VSWE system has excellent repeatability and show a proof-of-concept of its ability to map regional differences in and detect treatment-induced changes to the elastic modulus resulting from changes in tumour integrity.

## 2. Materials and Methods

### 2.1. Cell Culture

Human triple-negative luc-MDA-MB-231 LM2–4 breast cancer cells and luc-U87 MG human glioblastoma cells were authenticated by short tandem repeat (STR) profiling and tested negative for mycoplasma infection prior to tumour implantation. Cells were grown under aseptic conditions in Dulbecco’s modified Eagle’s medium (DMEM; Invitrogen, Life Technologies Corp., Carlsbad, CA, USA) supplemented with 10% (*v*/*v*) foetal bovine serum (PAN Biotech Ltd., Wimborne, UK).

### 2.2. Animals

Adult (5–6-week-old) female athymic NCr-Foxn1nu mice (*n* = 29, Charles River Ltd., Kent, UK) were used. Mice were housed in specific pathogen-free rooms in autoclaved, aseptic microisolator cages with a maximum of 5 animals per cage and allowed access to sterile food and water ad libitum. On the day of inoculation, cells were washed, trypsinised and counted before re-suspending them in a mixture of DMEM and Matrigel (Corning Ltd., St David’s, UK) (1:1) for injection. A total of 5 × 106 luc-MDA-MB-231-LM2-4 (referred to as MDA-MB-231 tumours from here on) or luc-U87 MG cells (referred to as U-87 MG tumours from here on) per mouse were injected subcutaneously in the right flank. Tumours were measured twice a week using callipers and were imaged when the tumours reached a volume of ~350 mm^3^. For imaging, anaesthesia was induced by an intraperitoneal 5 mL/kg injection of a combination of fentanyl citrate (0.315 mg/mL) plus fluanisone (10 mg/mL; Hypnorm, Janssen Pharmaceutical Ltd., Oxford, UK) and midazolam (5 mg/mL) (Roche Ltd., Welwyn Garden City, UK) and water (1:1:2). They were then secured sideways on a warm platform maintained at 38 °C, with tumours facing up.

Repeatability and kernel size optimisation study: initially, a cohort of mice (*n* = 5) with MDA-MB-231 tumours were imaged for three consecutive days using 3D-VSWE. This was to both test the repeatability of the set-up and measurements and to use the data to optimise the number of vibrational sources required (one or two shakers) and the kernel size used for shear wave speed calculation.

Therapeutic challenge: To assess the sensitivity of our approach to changes in tissue integrity, we chose to use a therapeutic challenge with ZD6126 (N-acetylcolchinol-O-phosphate), a vascular disrupting agent shown to induce central necrosis in a wide range of tumour models 24 h after a single dose of 200 mg/kg [28]. ZD6126 was formulated in 20% of 5% sodium carbonate and 80% phosphate-buffered saline and administered intraperitoneally. Mice with established MDA-MB-231 or U-87 MG tumours were imaged prior to and 24 h after treatment with either 200 mg/kg ZD6126 (*n* = 6 and *n* = 4, respectively) or vehicle alone (*n* = 5 and *n* = 4, respectively). One dataset at 1000 Hz using 2 shakers in the repeatability study and one dataset in the therapeutic challenge were excluded due to incomplete collection of data.

### 2.3. Vibrational Shear Wave Elastography (VSWE) Imaging Using Ultrasound

**Figure 1:**
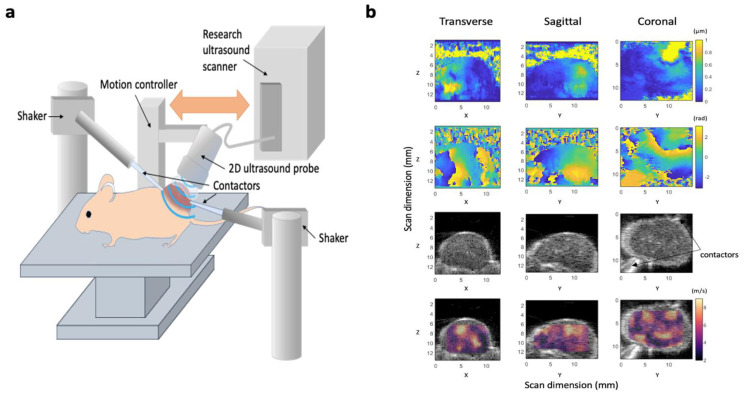
Design of our 3D-vibrational shear wave elastography (3D-VSWE) platform. (**a**) schematic showing the apparatus used to acquire vibrational shear wave elastography data. Two shakers were used as vibrational sources and were coupled to the tumours using carbon fibre rods and contactors. Shear wave fields were imaged using line-by-line focused imaging acquired with an L22-14vX linear array ultrasound probe and a Vantage system (both Verasonics Inc.). The probe was scanned superiorly in a step-and-shoot motion to collect 3D data. (**b**) Representative displacement amplitude maps (top row), phase maps (second row). B-mode image data (third row), and shear wave speed (c_s_) maps overlayed on B-mode data (bottom row) acquired using a vibrational frequency of 1000 Hz. Images are the central slices of 3D datasets in the transverse, sagittal and coronal planes; numbers give the scan dimensions in millimetres in the Z (axial), X (lateral) and Z (elevational) directions. Two contactors can be seen in the B-mode images, however, these data were acquired with only one vibrational source operating.

In our 3D-VSWE system, presented in Figure 1a, a signal generator was used to generate continuous sinusoidal signals of frequencies 700 Hz, 1000 Hz and 1200Hz to drive one or two vibrational sources (mini shakers, model 4810, Hottinger Bruel & Kjaer Ltd., Royston, UK). Shear waves were generated in tumours by coupling the two mini shakers via carbon fibre rods and custom-made Delrin^®^ contactors placed in physical contact with the skin overlying the tumour. The signal generator (Agilent 33120A, Agilent Scientific Instruments Inc., Santa Clara, CA, USA) was connected to a 2-channel 500 W audio amplifier (VLV-1000 Audio Intimidation Ltd., UK) that generated 1 or 2 narrow-band continuous signals of 700 Hz, 1000 Hz and 1200 Hz to drive the shakers. The lower frequency, 700 Hz, was chosen such that the shear wavelength would, on average, be less than the tumour dimensions, minimising the chance of whole tumour displacement as opposed to the desired shear-stress-induced deformation (i.e., generation of shear waves). For tumours less than 10 mm, the higher frequency of 1200 Hz allowed penetration of shear waves throughout the tumour. For stiffer tumours or those with greater width, shear waves may be attenuated and not be detectable across the entire tumour; hence we investigated using 2 vibrational sources to overcome shear wave attenuation at the higher frequencies. The mid-range frequency of 1000 Hz facilitated the comparison of shear wave speeds previously measured with MRE [8].

A line-by-line focused-beam ultrasound imaging sequence was used to detect shear waves, and the resulting shear wave fields were measured by moving a 1D array transducer across the tumour in a step-and-shoot manner to build up the 3-dimensional data. A Vantage 256 imaging system (Verasonics Inc., Kirkland, WA, USA) in conjunction with a high frequency (18.5 MHz nominal centre frequency) L22-14vX imaging probe (also Verasonics Inc.) was used. The Vantage system’s image reconstruction software was used to compute in-phase, and quadrature (IQ) sampled echo data allowing calculation and visualisation of B-mode images (see below). Shear wave oscillations were detected by repeat transmissions along individual A-lines with a pulse repetition frequency equal to 6 times the shear wave vibration frequency.

Radiofrequency data from the ultrasound scans were collected in the form of IQ data for analysis. Time points of scans, frequency of the vibrating shaker and number of shakers used during the scans formed variables for different scenarios of scanning, giving rise to a total of 18 datasets per mouse in the repeatability study and 12 datasets per mouse in the therapeutic challenge study. Given the step length of 0.2 mm in the Y-direction, a total of 50–75 images in the ZX plane were acquired for tumours depending on their Y-axis dimension (length), giving a scan length of 10–15 mm (Z = axial direction, X = lateral direction, Y = elevational direction). 

Shear wave phase maps (Figure 1b) show the phase of the displacement of the tissue caused by the propagation of shear waves through the tumour and, in addition to shear wave amplitude maps, can be visualized by the user in real-time to access the quality of the phase data. We have included a time series of phase maps to help illustrate shear wave propagation within the tumour (see Supplementary Material, Video S1). To generate shear wave phase maps, a 2D ultrasound phase-shift-based axial displacement estimator was applied to the IQ data to measure the local tissue axial displacement amplitude that was produced by the shear wave during each ultrasound pulse-to-pulse interval [29]. Fast Fourier transform of the tissue displacement versus time data was used to obtain the shear wave phase at each spatial location at the shear wave drive frequency. 

### 2.4. Shear Wave Speed Estimation

Shear wave speed was estimated by calculating the autocorrelation function of the 3D shear wave phase maps in the direction of local shear wave propagation. We employed a cube-shaped kernel-based autocorrelation approach similar to that used by Hoyt et al. [30]. A directional detector was used to extract 1D autocorrelation data aligned with the local shear wave propagation direction. A cosinusoidal fit to the autocorrelation data was used to estimate the shear wave wavelength, λ, and shear wave speed, c_s_, using the relationship: c_s_ = λ f, where f is the frequency. The dimension of the cuboid, or kernel, of data used to calculate the autocorrelation function directly influences VSWE spatial resolution and the ability to map heterogeneity of c_s_. Due to the use of a continuous source, we measured the phase velocity. 

### 2.5. Data Quality Metrics

**Figure 2:**
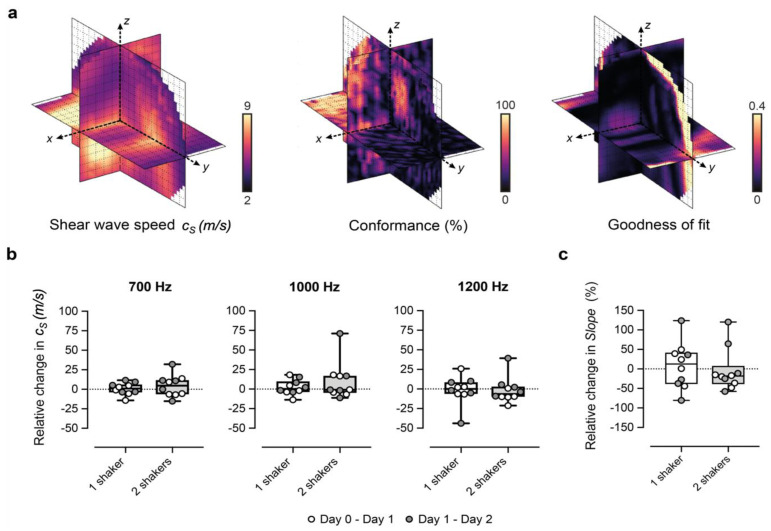
Performance of our 3D-vibrational shear wave elastography (3D-VSWE) platform. (**a**) shows orthogonal slices through 3D maps of c_s_, C and GoF across the tumour. Conformance is a measure of the percentage of the vibrational frequency detected at the vibrational source frequency and can be mapped to allow the user to assess the penetration of the shear waves during the positioning of the contactors. Similarly, the average Goodness of Fit (GoF) across the tumour allows real-time assessment of the quality of the data that can be obtained. (**b**,**c**) show the percentage change in spatial-median c_s_ and slope, respectively, between repeat measurements acquired 24 h apart. Data shown were generated using 2 mm kernels with 0.5 mm spacing and one vibrational source.

We devised two quality metrics (Figure 2a) which could be mapped and displayed to the user in real-time to aid with the placement of the contactors and the choice of vibrational frequency: 

(i) the Conformance enabled us to evaluate the quality of the detected shear wave field relative to vibrations at other frequencies and noise. C was calculated as the percentage of the total detected vibrational energy that was measured at the shear wave drive frequency:

$$C\left(\%\right)=\frac{100\;{\left|S_{f}\right|}^{2}}{\sum {\left|S_{i}\right|}^{2}}$$

where |*S_i_*| represents spectral amplitude components, and |*S_f_*| is the amplitude component at the shear wave frequency.

(ii) the Goodness of Fit (GoF), a measure of the mean residual error of the cosinusoidal fit to the autocorrelation function. GoF error indicates how well the shear wave phase and magnitude (over a cuboid kernel) conform to a travelling shear wave and provides a measure of the quality of the estimation of shear wavelength.

### 2.6. High-Frequency B-Mode Imaging

High spatial resolution B-mode images of tumours were obtained using a Vevo 770 and a 30 MHz transducer (Fujifilm Visualsonics Inc., Toronto, ON, Canada) prior to the acquisition of VSWE data for the improved alignment of histological sections with imaging data. Both Verasonics and Visualsonics transducers were positioned using custom-made holders such that the images from both systems could be easily co-registered.

### 2.7. Analysis of Ultrasound Datasets

IQ data obtained for a set of beamlines for a 3D ultrasound scan were first converted into B-mode images in the plane of the linear array for visualisation of tumours using simple log compression of the magnitude of the IQ data. For 3D analysis, B-mode images were imported to Microscopy Image Browser (MIB) for 3D segmentation of tumour outlines [31]. The 3D tumour outline was used to define a grid of measurement points for shear wave speed calculation inside the tumour. Shear wave speed calculation was performed for a kernel of tissue as described above. The size of the kernel and the percentage of overlap between the kernels determined the number of points that were placed on the image for analysis. The calculation was repeated, creating an (m × n × p) matrix of points across the 3D data set. The median value of all shear wave speeds estimated at all points was considered as the centroid of the c_s_ probability distribution for a tumour and has been used for data reduction to produce summary results for illustration in graphical form. Metrics that were used for quantitative analysis comprised C, GoF, c_s_ and Slope (slope of c_s_ versus shaker frequency). For the study of repeatability, C, GoF and c_s_ were calculated at various kernel sizes ranging from 0.5 mm to 3 mm to assess the effect of kernel size on these parameters. The within-subject coefficient of variation, CV_WS_ was calculated as previously described using the equation [32]:

$${CV}_{WS}\;(\%)\;=\;100\;\times \;([\Sigma (\Delta /m{)}^{2}]/2n{)}^{1/2},$$

in which m is the mean of the 2 paired repeat determinations of median c_s_ whose absolute difference is Δ, and the sum is taken over the *n* test–retest paired duplicate measurements. The CV_WS_ describes the reproducibility of a measurement device when repeat measurements are taken of the same subject, i.e., related samples. See for [33] further detail.

### 2.8. Histopathological Validation

After the final imaging, animals were euthanised with an overdose of sodium pentobarbital (Dolethal^®^). Tumours were excised, fixed in 10% neutral buffered formalin and embedded in paraffin. Aligned tumour sections (8 μm) were subsequently cut from the axial plane to reflect the central B-mode ultrasound images in the XZ direction and stained with hematoxylin and eosin (H&E) and for the murine vascular endothelial marker CD31 antibody (DIA-310, Dianova GmbH, Hamburg, Germany) in combination with Rat Histofine Max PO (414311F, Nichirei Bioscience Corp., Tokyo, Japan). Whole-slide images were digitized using a Zeiss Axioscan slide scanner (20× magnification, 0.46 μm resolution, Zeiss GmbH, Germany) and analysed using QuPath software [34]. The percentage of necrosis (large area of tissue damage) was calculated from regions of interest drawn manually by an experienced observer (YJ) blinded to the study parameters. This was expressed as the ratio of the area of the necrotic region to the total tumour area.

For the qualitative comparison of histological images and shear wave speed distribution, the digitized whole-slide H&E-stained images were visually aligned with the Visualsonics B-mode images (including rotation/reflection) using anatomical landmarks, including the shape of the tumour and attached skin. The observer was blinded to the processed 3D-VSWE-derived parametric shear wave speed maps, which were created by selecting the plane of shear wave speed measurement points at the centre of the tumour (i.e., the central m × n measurement points) using a 2 × 2 × 2 mm kernel for calculation. 

### 2.9. Statistical Analysis

Statistical analysis was performed using GraphPad Prism (GraphPad Software Inc., San Diego, CA, USA). Any significant differences in quantitative parameters were determined using the Wilcoxon test for the repeatability study, and any significant differences between groups were determined using the Mann-Whitney U-Test with a 5% level of significance.

## 3. Results

### 3.1. Optimization and Repeatability of Preclinical VSWE

**Figure A1:**
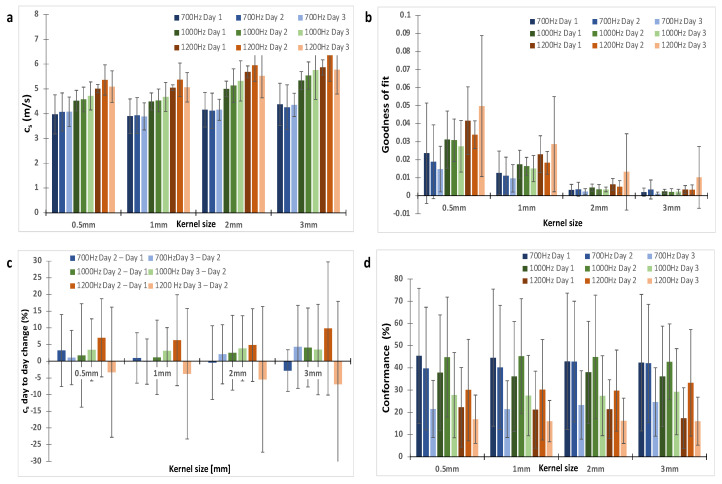
The choice of a 2 mm kernel dimension was based on the analysis of the shear wave speed (c_s_), Goodness of Fit and repeatability for kernel sizes of 0.5, 1, 2 and 3 mm. (**a**) shows shear wave speed was similar for all kernel sizes. (**b**) On average, GoF increased with vibrational frequency and decreased with kernel size. (**c**) the repeatability was assessed using the percentage change in c_s_ between repeat measurements acquired 24 h apart. (**d**) The conformance, which was the percentage of vibrational energy detected at the shear wave drive frequency given, varied little with kernel size as expected because it is assessed at the centre of the kernel. A kernel dimension of 2 mm offered good repeatability and small GoF error. The data shown are for one shaker. Trends were similar for data acquired with two shakers.

**Figure A2:**
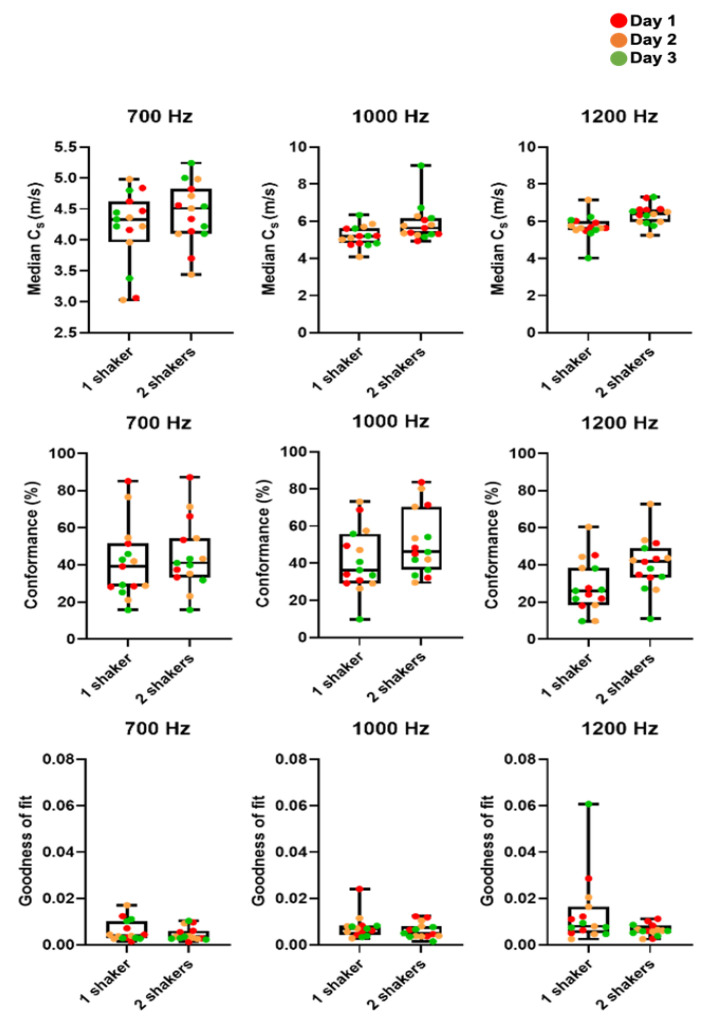
Box plots and whisker plots of c_s_, C and GoF in MDA-MB-231 tumours as measured on three consecutive days. We compared median c_s_, mean C and mean GoF for one and two vibrational sources (shakers) from all tumours (*n* = 5). There was no significant difference in C and GoF for two vibrational sources compared to one. Median c_s_ was greater for two shakers but not significantly so.

We optimised our 3D-VSWE system and tested its repeatability over three consecutive days in a cohort of five mice bearing subcutaneous tumour xenografts derived from the injection of MDA-MB-231 breast cancer cells. We found that most tumours could be scanned using a 10 mm scan length which took ~1.5 min. For three tumours, we used a 12 mm scan length (~1.7 min). For one tumour, we used a 15 mm scan length (~2.0 min). Allowing for time to restart the scan between frequencies, the total scanning time was just under 5 min for a 10 mm scan. Overall, we observed that c_s_ and C for the whole tumour remained similar for kernel sizes of dimensions 0.5, 1, 2, and 3 mm (Appendix A, Figure A1a,d). The GoF error was significantly less for 2 mm compared to 1 mm (Figure A1b). The mean change in c_s_ over 24 h was similar for all kernel sizes (Figure A1c). With respect to vibrational frequency, c_s_ and GoF both increased in magnitude with increasing frequency (700, 1000 and 1200 Hz), while C showed a reverse trend demonstrating that shear waves of higher frequency did not penetrate the tumour to the same depths as lower frequencies (Figure A1d) leading to greater noise in the displacement and phase data. Subsequent results have been obtained using a 2 mm kernel dimension. There were no significant differences in median c_s_, C or GoF values measured using one or two vibrational sources (Figure A2). Consistent with the viscoelastic properties of tissue [18], there was also a trend indicating increasing c_s_ values with increasing vibrational frequencies (Figure A1 and Figure A2). There was no significant difference in the whole measured tumour, c_s_, over 24 h. Example maps of c_s_, C and GoF and repeatability data for a 2 mm kernel size are shown in Figure 2. The within-subject coefficients of variation (CV_WS_) using one shaker were 5.2, 6.4 and 14.1% at 700, 1000 and 1200 Hz, respectively, and were greater when using two vibrational sources. CV_WS_ was poor for the slope of c_s_ vs frequency (>20%). Finally, kernel size had no effect on the within-subject coefficient of variation.

### 3.2. 3D-VSWE Can Detect Treatment-Induced Changes in Tumour Tissue Integrity

**Figure 3:**
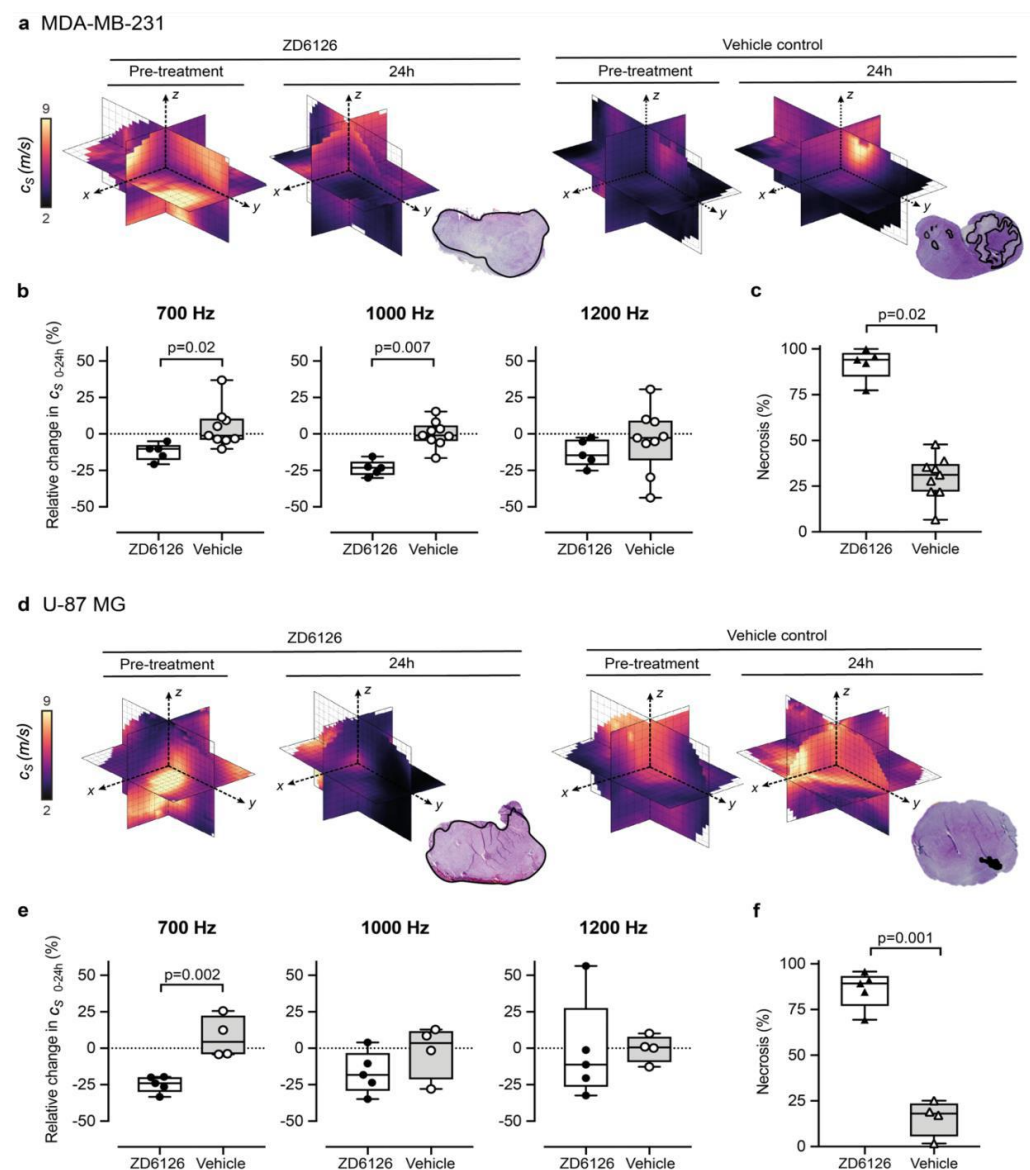
3D-vibrational shear wave elastography is sensitive to ZD6126-induced tissue damage. (**a**,**b**) show MDA-MB-231 human triple-negative breast cancer, (**d**,**e**) show U-87 MG human glioblastoma tumours. Representative 3D-VSWE derived parametric maps of c_s_ show a marked and global decrease in c_s_, 24 h following treatment with ZD6126 in both models, corroborated by the presence of extensive regions of haemorrhagic necrosis (outlined in black) visible on H&E-stained slide in stark contrast to the presence of a large area of viable tumour with a varied number of necrotic foci in the vehicle-control cohort. (**b**,**e**) show the quantitative analysis of the relative changes in spatial-median c_s_ values acquired at different frequencies, with either 200 mg/kg ZD6126 or vehicle, in both models (box plots show mean ± SD; Mann-Whitney test with 5% significant level). (**c**,**f**) show the quantitative analysis of the extent of necrosis quantified on the H&E-stained section.

**Figure A3:**
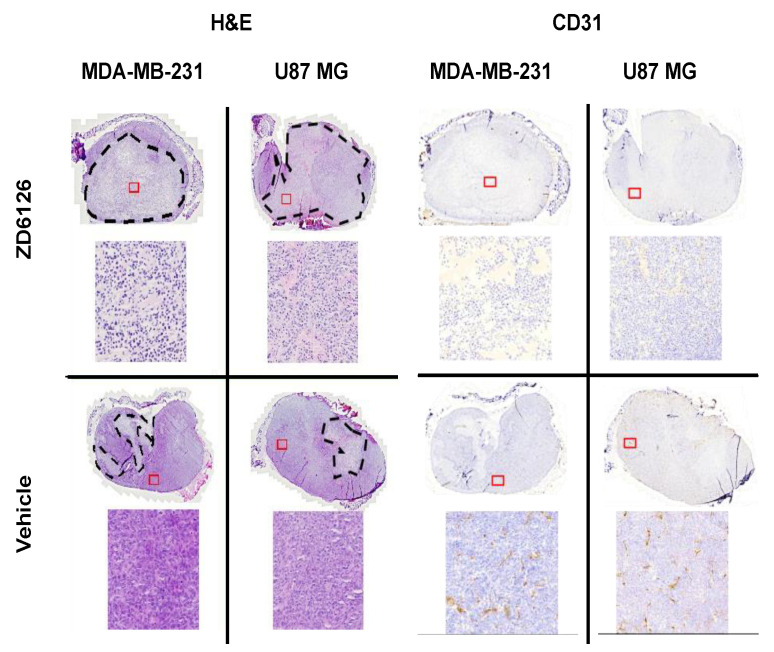
Representative histology sections stained with H&E and CD31 antibody. MDA-MB-231 and U-87 MG tumours treated with ZD6126 and vehicle are shown. The dashed black lines show the necrotic regions. Insets have been magnified to show necrotic and viable cell appearances. CD31 staining has been shown to demonstrate the functionality of ZD6126 on tumours.

We set out to show proof-of-concept of the ability of our 3D-VSWE preclinical system to detect therapy-induced changes in tissue elastic modulus resulting from the reduction in tissue integrity due to necrosis. To this end, we chose to measure changes in tumour c_s_ following the well-characterised pathophysiological response to the vascular disrupting agent ZD6126 in two xenograft models. We did not observe any change in shear wave speed dispersion, as indicated by the slope of the linear fit to tumour-median shear wave speed as a function of vibration frequency, from baseline in either treated or vehicle tumours in either model. In the vehicle cohort, there was no significant difference in measured whole tumour c_s_ over 24 h, with measured tumour-median values of c_s_ at baseline of 4.0 ± 0.7 m/s (*n* = 9) and c_s_ = 4.3 ± 0.3 m/s (*n* = 4) for MDA-MB-231 and U-87 MG tumours, respectively (mean ± SD). Twenty-four hours after treatment with ZD6126, an overall decrease in shear wave speed was apparent across the 3D parametric c_s_ maps of ZD6126-treated tumours, contrasting with the stable pattern seen in vehicle tumours (Figure 3a,d). This was corroborated by the quantitative analysis at 700 Hz showing a reduction in c_s_ of both tumour models (Figure 3b,e), resulting in significant differences in the relative changes in c_s_ over 24 h between the treated and vehicle cohorts in both models (U-87 MG: Δ c_s_ 24h = −24.7 ± 2.5 % vs 7.5 ± 7.1%, *p* = 0.002 and MDA-MB-231: Δc_s_ 24h = −12.3 ± 2.7 % vs 4.5 ± 4.7%, *p* = 0.02). These results were corroborated at 1000 Hz only for the MDA-MB-231 tumours (Δc_s_ 24h = −23.6 ± 2.4 % vs 0.0 ± 3.0% respectively, *p* = 0.007). Histopathological analysis of the tumours excised 24h after ZD6126 treatment showed an extensive central area of tissue damage (haemorrhagic necrosis) surrounded by a thin viable rim in all tumours—the characteristic pattern of response associated with successful treatment with a high dose of ZD6126 [8]. Quantitative analysis (Figure 3c,f) shows significantly higher mean necrosis area in ZD6126-treated cohort compared to vehicle cohort in both tumour models (U-87 MG: 86.0 ± 4.5 % vs 15.7 ± 5.0%, respectively, *p* = 0.001 and MDA-MB-231: 91.9 ± 3.9 % vs 29.6 ± 4.0%, respectively, *p* = 0.02). Representative images of ZD6126 and vehicle group tumours are shown in Appendix A Figure A3.

### 3.3. 3D-VSWE Can Provide a Map of Tumour Tissue Integrity 

**Figure 4:**
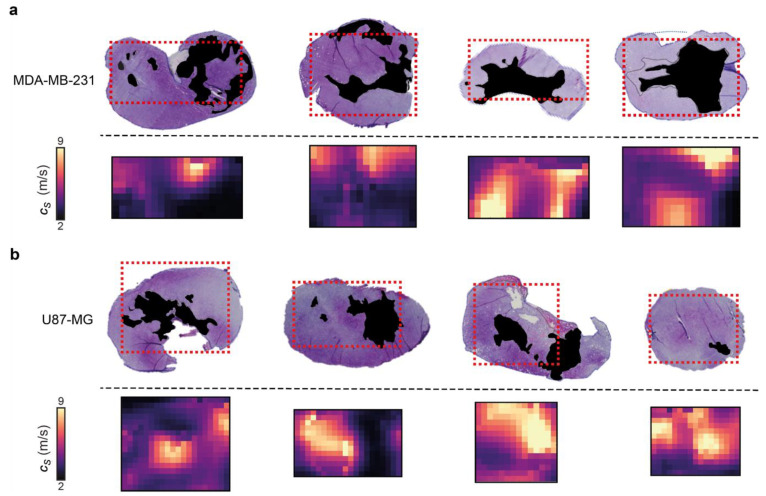
Comparison of 3D-VSWE derived parametric maps of shear wave speed c_s_ with aligned haematoxylin and eosin-stained histological sections. (**a**) shows MDA-MB-231 and (**b**) shows U-87 MG vehicle tumours. Necrotic regions in the tissue micrographs have been coloured black; in the c_s_ maps, the corresponding necrotic regions tend to have low c_s_, represented by dark purple and black. The spatial distribution of necrotic and viable tissue in vehicle tumours reveals that VSWE provides a good representation of the heterogeneous viable versus necrotic tissue within the tumour. Note that excised tissues are subject to shrinkage/deformation associated with pathological processing. Further, c_s_ maps are the central plane of the 3D data, which represents a 2 mm thick slice of tissue, whereas histological sections were about 8 µm thick. This may lead to partial volume effects; necrotic foci can have irregular distribution, i.e., tissue within one 8 µm slice may not give a good representation of tissues that lie at a distance on the order of a millimetre.

Control U-87 MG and MDA-MB-231 tumours present necrosis foci varying in number, location, shape and size, which enabled us to evaluate the ability of our 3D-VSWE to map spatial heterogeneity in tissue integrity. We performed a qualitative comparison of the spatial distribution of c_s_ with the distribution of necrosis and viable regions in aligned haematoxylin and eosin (H&E) stained tumour slides. The maps of c_s_ showed good visual agreement with the spatial distribution of viable (high c_s_) and manually-segmented regions of necrosis (low c_s_) in both tumour models, further highlighting the sensitivity of our 3D-VSWE to tissue damage and demonstrating the ability to resolve localised tissue damage (Figure 4).

## 4. Discussion

Beyond their potential for early disease detection, robust imaging methodologies enabling the non-invasive measure of tissue biomechanics and a comprehensive understanding of their topological variations are key to accelerating both the preclinical and clinical development of novel biomechanics-targeted therapeutic strategies, as well as providing more generic biomarkers of response to therapy, including extensive cell death, via the intricate relation between biomechanical properties and microstructure remodelling [35]. Tumour mechanical properties, as measured by elastography, are emerging as valuable imaging biomarkers for response assessment to chemotherapy and radiotherapy treatment, both preclinically and clinically [7,8,9,10,12,15,16,36,37,38]. Here, we have developed and implemented a dedicated small animal three-dimensional 3D-VSWE system which can reproducibly measure shear wave speed and its variation with frequency. 

Our development of 3D VSWE for preclinical imaging aimed to overcome the established limitations of 2D imaging and provide the whole-tumour mapping of c_s_ and increase the repeatability of measurements of c_s_, as has been shown clinically [39]. The within-subject coefficient of variation value for c_s_ measurement of our 3D-VSWE was found to be excellent for 700 and 1000 Hz (CV_WS_ < 6.5%), comparing favourably with the repeatability of other elastography techniques [8,37,40,41]. Median tumour c_s_ prior to treatment (measured using 700 Hz) was ~4 m/s, corresponding to an elastic modulus of ~48 kPa, which falls within previously reported ranges of elastic moduli of the central slice of subcutaneous tumours measured using a commercial clinical 2D ultrasound system [36,37,42]. The relative change in c_s_ measured with our 3D-VSWE upon treatment with ZD6126 in MDA-MB-231 and U-87 MG tumour xenograft corroborates those measured with MR elastography (Δc_s_ 24h ZD6126 = −15 ± 2 %) in human colon carcinoma SW620 xenograft at 7T (1000 Hz). 

Others have investigated ultrasound-based shear wave elastography for preclinical imaging. Some have used transient acoustic radiation forces impulses to generate shear waves to image tumours ex vivo [37,43] and in vivo [14,36,42,44,45], and murine liver in vivo [G] but these studies have so far been limited to 2D imaging and analysis. Nabavizadeh et al. [46] use a second transducer to generate shear waves that are less transient in nature than acoustic force impulses using 30-cycle bursts of ultrasound (duration 0.6 s). Using ultrafast imaging (1000) fps allows a 2D slice of tissue motion within a 2D slice. 

Although our technique uses a moving 1D array transducer (that collects 2D images) to build up a 3D volume, the analysis we perform is three-dimensional. Using a continuous vibrational source and repeatedly sampling the tissue motions at each transducer position, we build up a 3D map of tissue motion due to shear wave propagation. This enables us to identify the direction of the shear waves in three dimensions using our direction detector (as opposed to using directional filtering), and therefore measurements of shear wave speed are not subject to measurement bias from out-of-plane shear wave propagation [20].

Current MR elastography has higher spatial resolution than that achieved with our current system. However, the difference in acquisition time for one vibrational frequency (~1.5–2 min compared to 12 min for MRE), in addition to the established advantages of ultrasound over MRI in availability, footprint, and costs, make our 3D-VSWE an attractive alternative for high-throughput longitudinal monitoring of tumour biomechanics in preclinical studies in small animals. Another advantage of our 3D-VSWE method is that it can be tailored in terms of shaker frequency and position to suit tumours of different types (cell line, stage of growth, size, type of treatment, time after treatment, etc.). For example, in both tumour types studied here, there was a trend for increased variability and a smaller reduction in shear wave speed post-treatment for 1000 Hz and 1200 Hz compared to 700 Hz. It is important to note that 700 Hz may not be optimal for all types of tumours or tumour locations. The biomechanical properties of the tumour will influence shear wave attenuation, and therefore the ability to tune the vibrational frequency is essential. Stiffer tumour models may attenuate shear waves more rapidly, and therefore we have developed our technique to calculate and display representative maps of C and GoF (Figure 2a) during data acquisition, helping the user select the position of the contactor and the frequency of vibration to obtain the best shear wave field data. 

Following the use of external vibration sources in clinical reverberant field elastography [47], we have also looked at implementing two vibrational sources instead of one, although our aims were somewhat different. Rather than explicitly trying to generate a reverberant field, our aim was to increase the amount of vibrational energy detectable through the tumour to compensate for the rapid attenuation of shear waves at higher frequencies. Although we confirmed our hypothesis, we did not see a benefit in terms of repeatability or sensitivity of the technique to changes in shear wave speed and concluded that the use of one vibrational source only was appropriate for use with subcutaneous tumour models in mice. This has a practical benefit, as one source is easier to incorporate into the imaging set-up. For larger tumours, as might be expected in larger animals, or tumours that are less accessible, it is expected that lower frequencies or multiple sources may be required. When using multiple sources, the user should select the position (and frequency) of the sources bearing in mind that destructive interference of shear waves may reduce shear wave amplitude, reducing the ability to detect tissue motion due to shear wave propagation. Reducing shear wave amplitude will also reduce conformance, and therefore conformance maps may be used to help the user select the correct positions and frequencies to avoid this effect. For larger animals, in addition to lower frequency vibrational sources, lower frequency ultrasound transducers will also be required to provide the depth of penetration for shear wave detection. 

Clinically, similar approaches using vibrational sources have been demonstrated using matrix arrays [26,48]. Our study differs from these as it focuses on developing a device for use in the preclinical setting, which allows imaging at high ultrasound and shear wave frequencies that have the potential to provide high spatial resolution imaging. It is the first study that shows 3D ultrasound VSWE imaging in live mice and demonstrates that VSWE is sensitive to changes due to therapy. Additional novel aspects and differences to [26,49] include a novel shear wave direction detection method rather than direction filtering and the use of direct synchronisation of the 1D transducer array with the vibrational source signal generator allowing us to sample above the Nyquist limit with no need for additional processing to conduct phase alignment of sub-volumes. 

The ability to acquire scans rapidly means that multiple frequencies may also be used to measure multi-frequency shear wave dispersion, which is sensitive to the underlying tissue architecture, and molecular composition and is influenced by the solid/liquid state of the tissue [49]. Using 700, 1000, and 1200 Hz did not provide a reliable measure of shear wave dispersion, which may be due to the variable c_s_ values obtained at the higher frequencies. In addition to improvements in spatial resolution, the next steps include the investigation of the range and number of frequencies that allow us to reliably characterise viscous tumour moduli. 

Our method has limitations. In terms of spatial resolution, we identified that the kernel dimension of 2 mm provided the best fit (smaller GoF error) for the estimation of c_s_, as evidenced by the accurate representation of the spatial distribution of tissue shear wave speed. We have also shown that kernel sizes of 0.5 mm are feasible (Figure A1), but further work is required to improve the signal-to-noise ratio in characterising the shear wave field at the smaller kernel sizes to maintain an acceptable degree of variation in shear wave speed estimates. Noise reduction methods could potentially be implemented to improve the signal-to-noise ratio: sampling the shear wave field for a longer period or at an increased sampling rate may reduce noise in the shear wave displacement-time data, which in turn can reduce measurement variation. By implementing plane-wave imaging, the above suggestions could be attempted without compromising the speed of acquisition. Further improvements in spatial resolution could also be achieved by using a higher frequency transducer (our system currently uses 18.5 MHz) and wide aperture ultrasound focusing on more than one dimension, achievable with a 2D matrix array transducer [50]. However, these are not currently available at the high frequencies required for imaging mice. It should be noted that respiratory-induced tissue motion will effectively blur the shear wave speed map reducing spatial resolution. In this study, we used an anaesthetic that depressed respiration; if alternative injectable or gaseous anaesthetics are used, motion correction may be required to optimize spatial resolution. Future work will investigate the implementation of motion correction to avoid blurring due to physiological motion. 

## 5. Conclusions

We have developed a preclinical 3D vibrational elastography system for the rapid evaluation of tumour stiffness in vivo, which can quantify and map regional heterogeneity in tissue shear wave speed throughout the whole tumour. With the speed, availability and low cost of ultrasound, our 3D-VSWE system represents an invaluable tool to enable the non-invasive assessment of cancer therapies. These include therapies targeting the ECM and mechano-transduction to further understand how the biomechanical properties of tumours influence tumour progression, resistance to therapy, and their metastatic potential. 

## Data Availability

The data presented in this study are available in Supplementary Material.

## Supplementary Materials

The following supporting information can be downloaded at: https://www.mdpi.com/article/10.3390/cancers14194832/s1, Video S1: A time series of phase maps to illustrate shear wave propagation within the tumour.

## Appendix B

Excluded datasets: For the repeatability study, one dataset at 1000 Hz using two shakers was excluded as it gave shear wave speeds more than 9 m/s, which was ~100% greater than values obtained at 700 Hz and 1200 Hz. This was inconsistent with all other recorded datasets, in which 1000 Hz gave shear wave speeds higher than 700 Hz and lower than 1200 Hz. This dataset had the lowest conformance and greatest GoF error compared to the data obtained with two shakers at 700 Hz and 1200 Hz in the same imaging session. For the therapeutic challenge study, one mouse bearing an MDA-MB-231 tumour was excluded as endpoint histology showed none of the hallmarks of ZD6126 treatment such as central necrosis, reduced vascular density or presence of a large number of cells undergoing cell death.
